# Prevalence of normoalbuminuric renal insufficiency and associated clinical factors in adult onset diabetes

**DOI:** 10.1186/s12882-018-1001-7

**Published:** 2018-08-13

**Authors:** Polwatta Liyanage Gayani Chandima Liyanage, Sarath Lekamwasam, Thilak Priyantha Weerarathna, Dodanduwa Waduge Shyamal Yashodara Srikantha

**Affiliations:** 1 Department of Pharmacology ,Faculty of Medicine, University of Ruhuna, Galle, Sri Lanka; 2 Department of Medicine, Faculty of Medicine, University of Ruhuna, Galle, Sri Lanka

**Keywords:** Albuminuria, Chronic kidney disease, Diabetes

## Abstract

**Background:**

Microalbuminuria signifies the onset of diabetic nephropathy, but normoalbuminuric patients with diabetes who have a low Glomerular Filtration Rate (GFR) are not uncommon. The purpose of the study was to estimate the prevalence of such patients and to assess the clinical correlates.

**Methods:**

Cross-sectional study included patients with diabetes attending medical clinics at Teaching Hospital Galle. Diagnosis of albuminuria was made if urinary albumin excretion was > 30 mg/g of creatinine in two out of three samples. Patients were stratified into chronic kidney disease stages according to the estimated GFR (eGFR) calculated by Modification of Diet in Renal Disease (MDRD).

**Results:**

Mean (SD) age and duration of the disease of 456 (348 females) patients with diabetes were 60 (12) years and 10 (4) years. Sixty (13.2%) patients had low eGFR and 26.7% of them had normoalbuminuria. In the total sample, the proportion of patients with low eGFR and normoalbuminuria was 16 (3.5%). Among the patients with normoalbuminuria and low eGFR, 12.5% had retinopathy and none had any form neuropathy. When age, duration of disease, systolic and diastolic blood pressures, smoking, glycaemic control, presence of hypertension and ischaemic heart disease were included in binary logistic regression model, only age was found to be significant different (OR = 1.1, *P* = 0.03).

**Conclusion:**

A considerable proportion of adult diabetics are normoalbuminuric despite low eGFR. This limits the role of microalbuminuria as a screening tool to detect the onset of diabetic nephropathy. These patients do not exhibit distinct clinical features that facilitate identification of them using clinical information.

## Background

Of the long standing complications of diabetes, diabetic nephropathy (DN) has gained attention of clinicians and researches due to its progression to Chronic Kidney Disease (CKD) and End Stage Renal Disease (ESRD). Approximately 30% of the patients on renal replacement therapy have diabetes [1].

Chronic hyperglycemia and resulting advanced glycated end products in diabetes are the two most likely causative factors of DN [2]. Natural history of DN comprises of five stages; glomerular hypertrophy and hyperfiltration, renal structural changes without microalbuminuria, microalbuminuria with preserved renal function, significant proteinuria with progressive renal impairment and finally the development of ESRD [3]. Most accurate information on severity of DN is provided by histological evaluation of the affected kidneys.

The development of microalbuminuria is considered to be the first biochemical marker of diabetic nephropathy [4] while persistent albuminuria indicates overt nephropathy [5]. Renal insufficiency is characterized and staged reliably by calculating estimated glomerular filtration rate (eGFR) from an abbreviated equation from the Modification of Diet in Renal Disease (MDRD) study. CKD is diagnosed when e GFR is less than 60 ml/min/1.73m^2^ for at least 3 months duration [6]. It has been found that there is a good correlation between reduction of eGFR and degree of albuminuria in a proportion of patients with DN [7].

Although it is believed that DN begins with microalbuminuria and progresses to overt albuminuria before renal insufficiency occurs, studies have shown that a significant proportion of patients do not follow this set pattern and have low e GFR without albuminuria. This entity is called normoalbuminuric renal impairment (NARI) and in patients with type 2 diabetes it has a distinct clinical picture and is not clearly associated with poor glycaemic control [8]. The causation and pathophysiology of this distinct type of renal involvement in diabetes, however, has not been studied in detail in South Asian region. This cross-sectional study was aimed to estimate the prevalence of normoalbuminuric renal impairment and to study its clinical associations among patients with diabetes attending a tertiary care hospital in Southern Sri Lanka.

## Methods

This cross -sectional study included randomly selected patients more than 20 years of age with diabetes attending medical clinics at Teaching Hospital, Galle (THG). Pregnant or lactating women and patients with acute or chronic infections were excluded. Patients with diseases that can potentially influence renal function or albuminuria such as systemic lupus erythematosus, urosepsis, and nephrotic syndrome were also excluded.

**Fig. 1:**
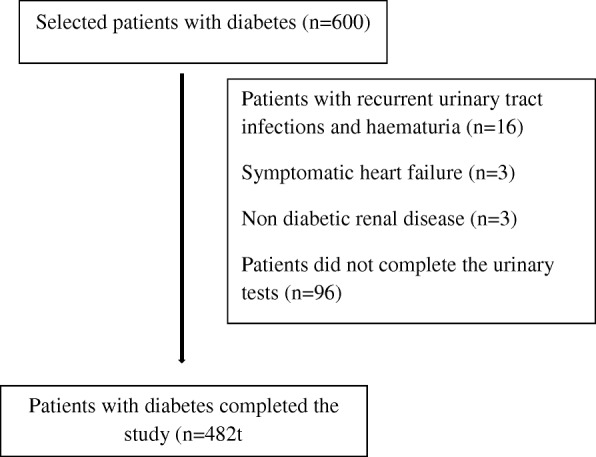
Flow chart of the study participants

Patients were identified using patient registries maintained in four medical clinics and out of them 600 patients were selected randomly and screened at the Department of Medicine on a selected date. After explaining the procedure an informed written consent was obtained from all patients (Fig. 1).

In the subjects selected, morning urine samples were collected and urine dipstick test was done to exclude ongoing urinary tract infections. (Urine collection was postponed if patient had fever, urinary symptoms suggestive of a urinary tract infection or menstruation). Only the urine samples negative for nitrates were stored at -80 °C for urine albumin analysis. Presence of albuminuria was tested by turbidimetric method [9]. Creatinine content in urine samples was measured by Jaffe reaction [10]. Each patient underwent testing of urine three times during the course of 3 months and categorization of microalbuminuria was confirmed if two out of three urine samples were positive for albumin (> 30 mg albumin/g of creatinine). Those who had albuminuria > 300 mg albumin/g of creatinine were diagnosed as having macroalbuminuria.

Identification of the presence of chronic kidney disease and its stage among the study population was done by e GFR values obtained using MDRD formula [6]. The degree of glycemic control was assessed by HbA_1_C level done by HPLC method [11].

A total of 600 patients with diabetes attending medical clinics at Teaching Hospital Karapitiya, Sri Lanka were selected randomly. Patients with type 2 diabetes who had recurrent urinary tract infections and haematuria (*n* = 16), symptomatic heart failure (*n* = 3), non-diabetic renal disease (n = 3) and those who did not complete the urinary tests (*n* = 96) were excluded from the study.

Anthropometric measurements such as weight, height, waist and hip circumferences were measured using standard scales and techniques. Blood pressure was measured in the sitting position in the right arm with a digital sphygmomanometer after 15 min of resting. Average of two readings which were taken 5 min apart was considered as the final blood pressure reading. Systolic blood pressure response to standing and diastolic blood pressure response to sustained handgrip were measured. Ankle Brachial Pressure Index (ABPI) was measured using mini doppler. It was classified as low ≤0.9, normal 0.9–1.3 and high > 1.3.

The validated Sinhala version of Rose Angina Questionnaire (RAQ) was administered to the subjects. This questionnaire was administered by a medical officer who had a prior knowledge on the rules applicable to filling the questionnaire. Angina and possible infarction were defined according to the World Health Organization (WHO) guidelines given with the original RAQ.

Retinopathy was detected by direct ophthalmoscopy using a Heine ophthalmoscope in a darkened room. In doubtful and difficult situations, the help of the consultant ophthalmologist were sought.

Diabetic Neuropathy Symptom (DNS) and Diabetic Neuropathy Examination (DNE) scores were obtained according to the guidelines. Score of more than 1 in DNS and more than 3 in DNE were considered having diabetic peripheral neuropathy. Semmes Weinstein Monofilament (SW-MF) 10 g was used to detect neuropathy according to the standard “yes/no” method of administration.

Further, we made an extensive search in the history and clinical examination to detect non-diabetic renal disease. In addition previous case records were examined. None of the patients included in the current study had evidence previous acute kidney injury or non-diabetic renal disease.

### Statistical analysis

All statistical analyses were performed using the Statistical Package for the Social Sciences. One way Analysis Of Variance (ANOVA) and unpaired t-test were used to compare continuous variables and Chi-square test was performed to compare categorical variables in different groups. Logistic regression analyses were performed to detect the determinants and associations of albuminuria and low e GFR. *P* < 0.05 was considered statistically significant.

## Results

The mean (SD) age of the study sample (*n* = 482) was 61 (11) years and 75% of them (*n* = 360) were females. Of the patients with diabetes studied, 286 (60.9%) had microalbuminuria and 24 (5.1%) had macroalbuminuria. The prevalence of diabetic nephropathy defined according to albuminuria (both micro and macro) was 66%.

Regarding the treatment, 369 (76%) of patients were on Angiotensin Converting Enzyme Inhibitor (ACEI) or Angiotensin Receptor Blocker (ARB). Percentage of ACEI or ARB treated patients in normoalbuminuric, microalbuminuric and macroalbuminuric patients were 68.8, 78.3 and 81.1% (*P* = 0.57) respectively.

Descriptive data of the patients participated in the study are shown in Table 1.

**Table 1:** Descriptive data of patients with diabetes included in the study (*n* = 482)

Factor	Percentage
Age (years)^a^	60.7 (10.5)
Gender (male)	24.9%
Retinopathy	21.4%
Angina according to the RAQ	23%
MI according to the RAQ	3.5%
ABPI	
Lowest to 0.9	11.9%
0.9 to 1.3	86.2%
> 1.3	1.9%
Hypertension	68.7%
Neuropathy	
DNS	19.5%
DNE	13.1%
Monofilament	39%
Biothesiometer	3.5%
Postural drop	23.4%
Handgrip	
Abnormal	31.7%

^a^Given mean (SD)

*MI* (Myocardial infarction), *RAQ* (Rose angina questionnaire), *ABPI* (Ankle brachial pressure index), *DNS* (Diabetic Neuropathy Symptom score), *DNE* (Diabetic Neuropathy Examination score)

One hundred and seventy four (42.9%) patients had eGFR < 60 ml/min and of them 43 (43/174, 24.7%) were normoalbuminuric. In the total sample, the proportion of patients with low eGFR and normoalbuminuria was 43 (10.8%) (Table 2).

**Table 2:** Number of patients with type 2 diabetes classified by CKD stages and albuminuria stages

	Normoalbuminura *N* = 134	Albuminuria *N* = 262
CKD 1–2 (*n* = 228) eGFR ≥ 60 ml/min	91	137
CKD 3 (*n* = 164) eGFR 30–59 ml/min	43	121
CKD 4 (*n* = 3) eGFR 15–29 ml/min	0	3
CKD 5 (n = 1) eGFR < 15 ml/min	0	1

*CKD* (Chronic kidney disease)

Comparison of clinical characteristics among patients with diabetes when considered both albuminuria and eGFR is shown in Table 3.

**Table 3:** Comparison of clinical characteristics among patients with diabetes when considered both albuminuria and eGFR

	Normoalbuminuric & eGFR > 60 *n* = 92	Normoalbuminuric & eGFR < 60 *n* = 43	Albuminuric & eGFR > 60 *n* = 138	Albuminuric & eGFR < 60 *n* = 127	*P* value
Retinopathy	3 (3.3%)	3 (7.0%)	43 (32.3%)	40 (33.1%)	< 0.001
Neuropathy	21 (23.1%)	17 (40.5%)	57 (41.6%)	64 (50.4%)	0.001
Low ABPI	6 (6.5%)	6 (14.0%)	18 (13.1%)	18 (14.4%)	0.56
Hypertension	55 (59.8%)	33 (76.7%)	89 (64.5%)	97 (76.4%)	0.03
Rose angina	17 (23%)	9 (30%)	32 (25.6%)	35 (29.2%)	0.77

Values are number (%)

*ABPI* (ankle brachial pressure index)

Table 4 shows a comparison of clinical characteristics in relation to nephropathy status according to albuminuria among patients with diabetes. Patients with albuminuria had a longer duration of diabetes when compared with normoalbuminuric patients (*P* < 0.05). Systolic blood pressure, age and poor glycaemic control were significantly higher among patients with albuminuria when compared with normoalbuminuric patients (*P* < 0.05). Smoking, Body Mass Index (BMI), Diastolic Blood Pressure (DBP), gender and Waist Hip Ratio (WHR), however, were not significantly different between patients with albuminuria and normoalbuminuria.

**Table 4:** Comparison of clinical characteristics between patients with and without albuminuria

Variable	Normoalbuminuria	Albuminuria	*P* value
Number (%)	160 (34%)	310 (66%)	
Age (years)	58 (11)	62 (10)	< 0.001
Proportion of males	36 (22.5%)	79 (25.6)	0.45
Duration of diabetes (years)	7.5 (2.7)	11.2 (4.6)	< 0.001
Proportion of Smokers	17 (10.6%)	40 (12.9%)	0.47
BMI (kg/m^2^)	23.7 (3.9)	23.7 (3.5)	0.84
WHR	0.87 (0.07)	0.88 (0.07)	0.4
SBP (mmHg)	128.2 (18.2)	135.7 (18.9)	< 0.001
Mean DBP (mmHg)	75.3 (9.5)	75.9 (10.4)	0.53
Proportion of poor glycaemic control	9 (7.3%)	166 (70%)	< 0.001

Data are mean (SD) or n (%)

*BMI* (body mass index), *WHR* (waist hip ratio), *SBP* (systolic blood pressure), *DBP* (diastolic blood pressure)

Regression analysis revealed that poor glycemic control and duration of diabetes were significant associations of albuminuria in patients with diabetes (Table 5). Although there were many variables associated with albuminuria, the regression model retained only poor glycaemic control and disease duration as significant associations of albuminuria.

**Table 5:** Association between clinical risk factors and albuminuria (either micro or macro)

Clinical risk factor	Odds ratio (95% CI)	*P* value
Age	0.99 (0.96 to 1.03)	0.87
Smoking	1.93 (0.57 to 6.52)	0.29
Duration of diabetes	1.35 (1.21 to 1.51)	< 0.001
SBP	1.01 (0.99 to 1.03)	0.32
DBP	0.99 (0.95 to 1.034)	0.74
Poor glycaemic control	31.44 (13.82 to 71.52)	< 0.001

*SBP* (systolic blood pressure), *DBP* (diastolic blood pressure)

For the next analyses, nephropathy was defined based on the e GFR regardless of albuminuria. In these patients comparison of clinical characteristics with the CKD stages is shown in Table 6.

**Table 6:** Comparison of clinical characteristics among patients with diabetes in relation to CKD stages

	CKD stages		*P* value
	Stage 1 & 2 e GFR > 60	Stage 3,4 & 5 e GFR < 60	
Number (%)	231 (57%)	174 (43%)	
Age (years)	56 (10)	67 (8)	< 0.001
Proportion of males	60 (26)	40 (23)	0.28
Duration of diabetes (years)	9 (4)	11 (4)	0.001
Proportion Smokers	36 (16)	13 (7)	0.009
BMI (kg/m^2^)	24.7 (3.5)	22.3 (3.4)	< 0.001
WHR	0.88 (0.06)	0.86 (0.07)	0.04
SBP (mmHg)	132 (17)	136 (20)	0.03
DBP (mmHg)	76 (10)	76 (10)	0.96
Proportion of poor glycaemic control (HbA1c > 7.5%)	86 (47)	58 (48%)	0.46

Data are means (SD) or n (%)

*CKD* (Chronic kidney disease), *BMI* (body mass index), *WHR* (waist hip ratio), *SBP* (systolic blood pressure), *DBP* (diastolic blood pressure)

Regression analysis revealed that glycemic control and duration of diabetes were significant associations of albuminuria in patients with diabetes (Table 5). Although there were many variables associated with albuminuria, the regression model retained only poor glycaemic control and disease duration as significant associations of albuminuria.

In the regression analysis, smoking and age were the significant determinants of low e GFR (Table 7).

**Table 7:** Association between clinical risk factors and reduced eGFR (eGFR < 60)

Clinical risk factor	Odds ratio (95% CI)	*P* value
Age	1.14 (1.1 to 1.18)	< 0.001
Smoking	5.21 (1.84 to 14.7)	0.002
Duration of diabetes	0.99 (0.93 to 1.06)	0.86
SBP	0.99 (0.97 to 1.01)	0.35
DBP	1.02 (0.98 to 1.05)	0.32
Glycaemic control	1.1 (0.61 to 1.98)	0.68

*SBP* (systolic blood pressure), *DBP* (diastolic blood pressure)

## Discussion

Results of this study reveal that among patients with renal insufficiency (e GFR < 60 ml/min/1.73m^2^) 26.7% were normoalbuminuric. According to several cross-sectional and cohort studies, it has been proven that the prevalence of NARI is not an infrequent finding among patients with diabetes [11]. The prevalence of NARI varies from 14 to 57% in cross- sectional studies carried out in various countries [11]. Our finding on prevalence of NARI is consistent with the most of the previous studies conducted in other countries such as in Australia and the USA where prevalence of NARI is 23.2% and 27% respectively [12]. Further, So et al. in 2006 reported prevalence of NARI as low as 14% in 4421 patients he studied [13].However, another study from Japan has reported a higher prevalence of NARI with, 51.8% of patient with low eGFR (< 60%) were normoalbuminuric [14].

The finding that NARI was less strongly associated with other microvascular complications of diabetes was also previously reported. A study from Italy reported that 76.5% of patients with NARI had no diabetic retinopathy [8]. It has been reported that 75.6% patients with NARI to be free of other microvascular complication [4]. Furthermore, the same authors reported that 63.4% of them to have no diabetic nephropathy or neuropathy. This is also consistent with our findings in which 96.7% of patients with NARI had no diabetic retinopathy and 76.9% had no neuropathy.

According to the data, the prevalence of albuminuria among total sample was 66% (95% CI: 61.7 to 70.2) with microalbuminuria was 60.9% (*n* = 286) while macroalbuminuria prevalence was 5.1% (*n* = 24).

Among total sample, the prevalence of low eGFR (< 60%) was 42.9% (*n* = 174) and age and smoking were the factors related to low eGFR. Other microvascular complication namely, retinopathy and neuropathy were associated with albuminuria but not with low eGFR.

It has been found that normoalbuminuric renal insufficiency is associated with several characteristics other than its association with low prevalence of microvascular complications of diabetes. They include female predominance, lower HbA1c, shorter duration of diabetes and lower prevalence of hypertension [15].

One may consider normoalbuminuric renal insufficiency as the forerunner of microalbuminuric renal insufficiency. In support of this, An J et al. found the prevalence of normoalbuminuric renal insufficiency to decrease with the increased duration of diabetes and advanced stage of retinopathy [15]. It is possible that some patients develop renal insufficiency with no albuminuria initially and then progress to microalbuminuria renal insufficiency as the disease progresses. This may question the use of microalbuminuria as the biochemical hallmark of diabetic nephropathy in clinical practice.

Our data were obtained from a hospital based sample of adult patients with type 2 diabetes, showed a higher prevalence of albuminuria. Although few studies have reported similar figures of albuminuria, most of the studies, both hospital based and population based, have shown lower prevalence of albuminuria. A population-based study from India which reported 29.1% prevalence of albuminuria, but the mean age of the patients was 52 years, about 9 years younger than our study sample [16]. This could be due to several reasons. These include variations in study population, study design and definition of the outcome variable. Compared to other studies our subjects were older and had longer duration of the disease. Higher mean age of the patients in our study could be a reason for the high prevalence of microalbuminuria since albuminuria increases with age. Older patients are likely to have a disease for a longer period, hence more likely to have developed long term complications of diabetes. Further, Unnikrishnan in his study considered presence of diabetic retinopathy in to the definition of diabetic nephropathy [16]. This might also be a reason for getting a less prevalence of albuminuria compared to our study.

Higher prevalence of other types of kidney disease such as chronic kidney disease of unknown origin (CKDu) in the general population in our country may also have contributed to the seemingly high prevalence of albuminuria in this study sample.

## Conclusion

A considerable proportion of patients with diabetes are normoalbuminuric despite low eGFR. This limits the role of microalbuminuria as a screening tool to detect the onset of diabetic nephropathy. These patients do not exhibit distinct clinical features that facilitate identification of them using clinical information.

## Abbreviations

ABPI: Ankle Brachial Pressure Index
ACEI: Angiotensin Converting Enzyme Inhibitor
ANOVA: Analysis of Variance
ARB: Angiotensin Receptor Blocker
BMI: Body Mass Index
CKD: Chronic Kidney Disease
CKDu: Chronic kidney disease of unknown origin
DBP: Diastolic Blood Pressure
DN: Diabetic nephropathy
DNE: Diabetic Neuropathy Examination
DNS: Diabetic Neuropathy Symptom
eGFR: estimated GFR
ESRD: End Stage Renal Disease
GFR: Glomerular Filtration Rate
HPLC: High performance liquid chromatography
MDRD: Modification of Diet in Renal Disease
MI: Myocardial infarction
NARI: Normoalbuminuric renal impairment
RAQ: Rose Angina Questionnaire
SW-MF: Semmes Weinstein Monofilament
THG: Teaching Hospital, Galle
WHO: World Health Organization
WHR: Waist Hip Ratio
